# Descriptive model for the prediction of motion direction from spike trains of ON-OFF directional selective retinal ganglion cells

**DOI:** 10.1186/1471-2202-15-S1-P124

**Published:** 2014-07-21

**Authors:** Aurel V Martiniuc, Victor Bocos-Bintintan, Florian Röhrbein, Alois Knoll

**Affiliations:** 1 Department of Computer Science, Technical University Munich, Garching, 85748, Germany; 2 Faculty of Environmental Science & Engineering, Babeş-Bolyai University, Cluj-Napoca, 400429, Romania

## 

**Figure 1:**
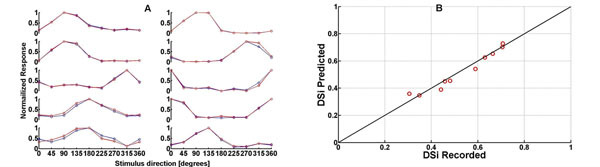
**A.** Normalized tuning curves for 10 ON-OFF DSRGCs. Recorded responses (red plots) and predicted responses (blue plots) are indicating the same direction of stimulus motion. **B.** DSis for predicted responses are slightly underestimating the DSis for recorded same ON-OFF DSRGCs in most of the cases.

A descriptive model to characterize a physiological property called *receptive field*, which is fundamental in deciphering how particular neurons encode the incoming visual stimulus, was built to accurately predict the direction of stimulus motion based on recorded directional selective retinal ganglion cells (DSRGCs) neural response. Briefly, building the model, space-time inseparability of the receptive fields of ON-OFF DSRGCs together with additional static non-linearity provided the principal key. The main steps we used were: analysis of the extracellularly recorded data from ON-OFF DSRGCs stimulated with white noise stimulus; calculation of spike triggered average in response to white noise stimulus and extracting the kernel; finding the optimal kernel by adjusting the estimated firing rate to the recorded firing rate; analysis of the extracellularly recorded data using new stimulus, consisting in drifting grating bars moving 45 degrees apart over the RFs of the ON-OFF DSRGCs; construction of the linear model; adding static non-linearity (i.e. spike threshold and saturation); comparing predicted data with recorded data. Similar methodology was used to test different scientific hypothesis [1], [2]. The optimal kernel needed to describe direction selectivity of ON-OFF DSRGCs was first extracted using white noise stimulus and produces an estimate of the firing rate that is a linear filter of the stimulus to which a nonlinear function was added. This is a function of the linear filter value instantaneously evaluated at the time of the rate estimation. Half wave rectification and saturation limit negative or unrealistically large values of the function. Static nonlinearity is then used to introduce firing thresholds into estimates of neural responses of ON-OFF DSRGCs. The direction of stimulus motion was accurately predicted for different preferred directions of the recorded cells by our model, as exemplified in Figure 1. However, the degree of direction selectivity (DSi) calculated from the tuning curves [3] was not 100% predicted. This slight underestimation of the direction selectivity degree (see Figure 1.B) suggests that for complex tasks, such as direction of stimulus motion, higher order non-linearity should be taken into account to improve the model performance. Intrinsic properties of ON-OFF DSRGCs like bursting or paired spiking activity can presumably improve the model accuracy. These results are in accordance with other scientific work related to different types of neurons and to different stimuli [1], [2].
